# Raffinose Family Oligosaccharides: Friend or Foe for Human and Plant Health?

**DOI:** 10.3389/fpls.2022.829118

**Published:** 2022-02-17

**Authors:** Dinakaran Elango, Karthika Rajendran, Liza Van der Laan, Sheelamary Sebastiar, Joscif Raigne, Naveen A. Thaiparambil, Noureddine El Haddad, Bharath Raja, Wanyan Wang, Antonella Ferela, Kevin O. Chiteri, Mahendar Thudi, Rajeev K. Varshney, Surinder Chopra, Arti Singh, Asheesh K. Singh

**Affiliations:** ^1^Department of Agronomy, Iowa State University, Ames, IA, United States; ^2^VIT School of Agricultural Innovations and Advanced Learning, Vellore Institute of Technology, Vellore, India; ^3^Division of Crop Improvement, ICAR-Sugarcane Breeding Institute, Coimbatore, India; ^4^School of Biosciences and Technology, Vellore Institute of Technology, Vellore, India; ^5^International Center for Agricultural Research in the Dry Areas, Rabat, Morocco; ^6^Faculty of Sciences, Mohammed V University of Rabat, Rabat, Morocco; ^7^Ecosystem Science and Management, Penn State University, University Park, PA, United States; ^8^Department of Agricultural Biotechnology and Molecular Biology, Dr. Rajendra Prasad Central Agricultural University, Pusa, India; ^9^Centre for Crop Health, University of Southern Queensland, Toowoomba, QLD, Australia; ^10^International Crops Research Institute for the Semi-Arid Tropics, Patancheru, India; ^11^State Agricultural Biotechnology Centre, Crop Research Innovation Centre, Food Futures Institute, Murdoch University, Murdoch, WA, Australia; ^12^Department of Plant Science, Penn State University, University Park, PA, United States

**Keywords:** α*-galactosides*, flatulence, *galactinol synthase*, prebiotic carbohydrates, grain legume crops

## Abstract

Raffinose family oligosaccharides (RFOs) are widespread across the plant kingdom, and their concentrations are related to the environment, genotype, and harvest time. RFOs are known to carry out many functions in plants and humans. In this paper, we provide a comprehensive review of RFOs, including their beneficial and anti-nutritional properties. RFOs are considered anti-nutritional factors since they cause flatulence in humans and animals. Flatulence is the single most important factor that deters consumption and utilization of legumes in human and animal diets. In plants, RFOs have been reported to impart tolerance to heat, drought, cold, salinity, and disease resistance besides regulating seed germination, vigor, and longevity. In humans, RFOs have beneficial effects in the large intestine and have shown prebiotic potential by promoting the growth of beneficial bacteria reducing pathogens and putrefactive bacteria present in the colon. In addition to their prebiotic potential, RFOs have many other biological functions in humans and animals, such as anti-allergic, anti-obesity, anti-diabetic, prevention of non-alcoholic fatty liver disease, and cryoprotection. The wide-ranging applications of RFOs make them useful in food, feed, cosmetics, health, pharmaceuticals, and plant stress tolerance; therefore, we review the composition and diversity of RFOs, describe the metabolism and genetics of RFOs, evaluate their role in plant and human health, with a primary focus in grain legumes.

## What Are Raffinose Family Oligosaccharides?

Raffinose family oligosaccharides (RFOs) are soluble carbohydrates ranked next to sucrose in their distribution in higher plants (French, 1954; Keller and Pharr, 1996). They are abundant in the seed of many crops, particularly in the legume family, e.g., soybean (*Glycine max*), lentil (*Lens culinaris*), and chickpea (*Cicer arietinum*). They are also present in roots and specialized storage organs such as tubers and leaves. For example, the RFOs concentration is up to 25–80% of their dry weight in tubers of Chinese artichoke (*Stachys sieboldii*) and photosynthesizing leaves of a common bugle (*Ajuga reptans*) (Bachmann and Keller, 1995; Tahir et al., 2011). However, due to the lack of α*-galactosidase* to degrade RFOs (Calloway and Murphy, 1968; Han and Baik, 2006), they are neither absorbed nor hydrolyzed in the upper gastrointestinal tract of humans and get accumulated in the large intestine of the human digestive system. Eventually, the α*-galactosides* undergo microbial fermentation by colonic bacteria resulting in hydrogen, methane, and CO_2_ production – major components of flatulent gases (Singh, 1985). RFOs are indigestible and cause flatulence in humans (Gupta, 1987; Van den Ende, 2013; Sengupta et al., 2015; Gangl and Tenhaken, 2016). Expulsion of these gases causes severe abdominal discomfort such as abdominal rumblings, cramps, diarrhea, and nausea (Sosulski et al., 1982; Kennedy et al., 1985; Lee et al., 2007).

**FIGURE 1:**
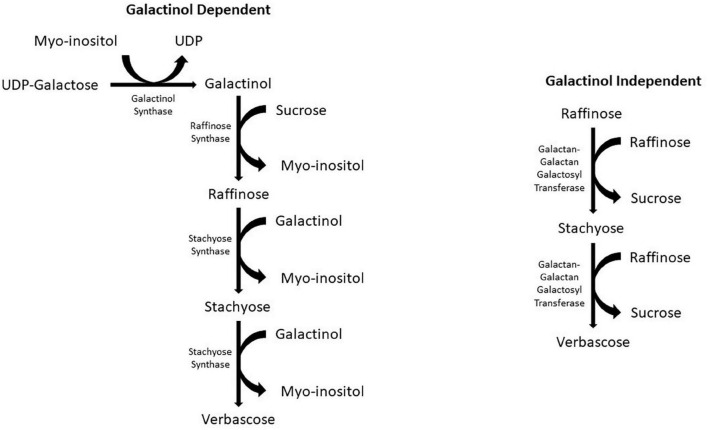
Galactinol dependent and independent biosynthetic pathways for RFO biosynthesis in crop plants.

The production of galactinol initiates the biosynthesis of RFOs by *galactinol synthase*, which catalyzes the galactosyl residue from UDP-D-galactose to myoinositol (Peterbauer and Richter, 2001; Panikulangara et al., 2004). Two pathways have been identified as RFO biosynthetic pathways (Figure 1). The first pathway, and the more common of the two, is galactinol dependent. This pathway starts with UDP-galactose and myoinositol as precursors. *Galactinol synthase* catalyzes the transfer of a galactosyl moiety from UDP-galactose to myoinositol, forming galactinol (Peterbauer and Richter, 2001). The next step in the pathway is catalyzed by the *raffinose synthase*, which transfers a galactosyl moiety from the previously formed galactinol to sucrose, forming raffinose, the first RFO in the biosynthetic pathway (Peterbauer et al., 2002a). Following the formation of raffinose, the larger RFOs stachyose and verbascose can be formed. The formation of stachyose is catalyzed by *stachyose synthase*, similar to the formation of raffinose; a galactosyl moiety is transferred from galactinol. During this reaction, raffinose is the acceptor instead of sucrose. The verbascose formation is also similar, except stachyose is now the galactosyl acceptor. The enzyme responsible for catalyzing this reaction has yet to be recognized (Lahuta et al., 2010); however, in peas (*Pisum sativum*), *stachyose synthase* is used as a multi-functional enzyme in synthesizing both stachyose and verbascose (Peterbauer et al., 2003).

The second RFO biosynthetic pathway is galactinol independent (Figure 1). This pathway is less common than the first and has only been reported in two species of the Lamiaceae family: *Ajuga reptans* and *Coleus blumei* (Bachmann and Keller, 1995; Gilbert et al., 1997). This pathway can only produce the larger RFOs of stachyose and verbascose. To form these RFOs, the enzyme *galactan-galactan galactosyl transferase* (*GGT*) catalyzes the transfer of a galactosyl moiety from one RFO, such as raffinose, to another RFO. In the formation of stachyose, two raffinose molecules act as donors and acceptors to form a single stachyose. Verbascose is formed by *GGT* catalyzing the transfer of galactosyl from raffinose to stachyose. The *GGT* enzyme has only been found in the vacuoles of leaves (Bachmann and Keller, 1995; Peterbauer et al., 2002b).

Raffinose family oligosaccharides can be reduced at varying degrees based on the food preparation involved. Food processing techniques including soaking, germination, decortications, fermentation, cooking, and use of enzymes such as α*-galactosidase* (which can catalyze the hydrolysis of RFO) can significantly increase the level of soluble dietary fiber fraction, reduce the levels of α*-galactosides* and hence enhance the digestibility of the food (Jood et al., 1985; Egbe and Akinyele, 1990; Aguilera et al., 2009). However, these food processing methods are time-consuming and lead to loss of nutrients and sometimes have consumer acceptability issues. Therefore, alternative approaches from a breeding perspective have been used to select cultivars with a low level of raffinose and stachyose (Obendorf and Górecki, 2012; Redekar et al., 2020) or inhibiting *galactinol synthase* activity (Bock et al., 2009) and over-expression of α*-galactosidase* in seeds by genetic manipulation (Polowick et al., 2009). Screening legumes for low RFOs contents has been carried out in many species such as chickpea (Raja et al., 2015; Gangola et al., 2016), lentil (Tahir et al., 2011, 2012), pea (Peterbauer et al., 2003), and soybean (Blackman et al., 1992; Dierking and Bilyeu, 2008; Obendorf and Górecki, 2012). Many breeding programs of grain legumes aim to decrease the content of antinutritional factors via genetic means to a safe extent to increase the level of grain legumes in human and animal diets.

Moreover, RFOs have recently been reported to have a beneficial effect on the gut microflora. Therefore, they are recommended in human diets to prevent cancer in the digestive tract (Van den Ende, 2013). The oligosaccharide family of raffinose has a wide range of predicted functions. In addition, being a form of carbohydrate storage and transport, the raffinose members play an important role in abiotic stresses, such as high salinity and drought (Taji et al., 2002; Nishizawa-Yokoi et al., 2008; Dobrenel et al., 2013; Van den Ende, 2013). Due to their membrane-stabilizing, antioxidant properties, and perhaps expected signaling roles, RFOs are emerging as crucial molecules during stress responses in plants (Van den Ende, 2013). We aim to comprehensively review the literature about RFOs and their role in human and plant health with this background.

## Raffinose Family Oligosaccharides Diversity in Crops

Raffinose family oligosaccharides are well documented in many cereals, pulses, fruits, and vegetables (Vidal-Valverde et al., 1993; Frias et al., 1994; Andersen et al., 2005; Wang and Daun, 2006; Huynh et al., 2008). The below table summarizes the genetic variability of different oligosaccharide components present in the grain legumes (Table 1). Range and mean values of each RFO component were presented, wherein some authors reported as range and others reported as mean.

**TABLE 1:** Variability of oligosaccharides present in the various grain legumes.

Crop	No of genotypes	Unit (dry matter)	Sucrose	Raffinose	Stachyose	Verbascose	Ciceritol	Total α*-galactosides*	References
Peas	18	g kg^–1^	11.6–25.4	4.1–10.3	10.7–26.7	0.0–26.7	–	22.6–63.4	Vidal-Valverde et al., 2003
	–	%	2.3–2.4	0.3–0.9	2.2–2.9	1.7–3.2	–	5.3–8.7	Asif et al., 2013
	1 (wild type)	g 100 g^–1^	1.8	0.6	1.5	2.2	–	4.3	Jones et al., 1999
	1	%	2.8	1.2	3.8	4.6	–	9.6	Sosulski et al., 1982
Soybean	241	mg g^–1^	46.8	8.3	31.7	–	–	40.0	Hou et al., 2009a
	195	g 100 g^–1^	6.1	1.0	2.3	–	–	3.3	Hymowitz and Collins, 1974
	20 (high protein-1991)	g kg^–1^	36.0	9.3	43.6	–	–	52.9	Hartwig et al., 1997
	20 (high oil-1991)		46.2	10.7	39.9	–	–	50.5	
	Soybean	mg g^–1^	48.1	6.2	38.4	1.6	–	46.2	Grieshop et al., 2003 (Processing plant #1)
	Soybean meal		69.4	13.3	57.2	2.3	–	72.8	
	Williams	%	5.6	0.9	4.1	–	–	10.6	Kennedy et al., 1985
	Forrest		6.0	0.9	3.9	–	–	10.8	
	Big Jule		7.7	0.9	3.9	–	–	12.5	
	4	μmol g^–1^	165.2	24.2	70.5	–	–	94.7	Hitz et al., 2002
	20	mg g^–1^	4.5	0.9	3.4	–	–	4.3	Hou et al., 2009b
	5	%	7.2	3.6	4.6	–	–	8.2	Giannoccaro et al., 2008
	148	mmol 100 g^–1^	–	0.6–2.5	2.1–7.1	–	–	2.7–9.6	Kumar et al., 2010
	20	%	6	0.7	4.1	–	–	3.9–5.3	Trugo et al., 1995
	1 (local market)	mg g^–1^	-	60.1	35.0	Not detectable	Not detectable	95.1	Han and Baik, 2006
	1	%	6.4	1.2	2.9	–	–	4.6	Sosulski et al., 1982
Chickpea	–	%	33.7	7.7	27.3	–	–	35.0	Lineback and Ke, 1975
	Desi-8 Kabuli-7	g 100 g^–1^	–	0.4–0.7 0.4–0.6	1.1–1.9 0.8–1.4	–	–	1.5–2.6 1.2–2.0	Singh et al., 1982
	1 (cv. Castellano)	g 100 g^–1^ (Wet basis)	2.3	0.6	1.2	–	2.8	6.9	Sánchez-Mata et al., 1998
	1 (cv. Pedrosillano)		1.1	0.6	0.7	–	2.5	4.9	
	A batch of 10 kg from the market	g 100 g^–1^	1.9	1.5	2.6	0.2	–	6.1	Alajaji and El-Adawy, 2006
	Raw	%	4.3	1.0	2.8	Traces	–	3.8	Åman, 1979
	Germinated (72 h)		4.9	0.3	0.7	–	–	1.0	
	1 (cv. Dwelly)	mg g^–1^	–	50.2	27	Not detectable	67.7	144.9	Han and Baik, 2006
	1	g kg^–1^	15.2	3.2	17.7	–	27.6	48.5	Aguilera et al., 2009
	213	mg g^–1^	3.6–54.1	0.2–15.1	2.8–59.4	–	4.4–90.1	7.4–164.6	Ramadoss et al., 2015
	1 (sample 171)	%	2.4	0.8	3.1	–	4.8	8.7	Xiaoli et al., 2008
	1	%	2.7	0.5	1.7	0.1	–	5.5	Sosulski et al., 1982
Lentil	4	%	1.7–2.5	0.3–0.5	1.7–2.2	0.4–0.7	–	2.4–3.4	Wang and Daun, 2006
	–	%	1.8–2.5	0.4–1.0	1.9–2.7	1.0–3.1	–	3.3–6.8	Asif et al., 2013
	143	mg g^–1^	208–1010	–	–	–	–	508–2167	Johnson et al., 2021
	1 (1994 harvest)	g 100 g^–1^	1.0	0.8	1.6	–	1.4	3.8	Sánchez-Mata et al., 1998
	1 (1995 harvest)		1.4	0.7	1.7	–	1.8	4.2	
	1 (cv. Pardina)	mg g^–1^	–	28.6	24.6	3.9	38.6	95.5	Han and Baik, 2006
	1 (cv. Crimson)		–	37.0	28.8	7.2	50.0	122.9	
	1	%	3.4	0.3	1.5	0.5	–	4.1	Sosulski et al., 1982
Faba bean	1	%	2	0.2	0.7	1.5	–	2.7	Sosulski et al., 1982
	1	g 100 g^–1^	-	0.3	1.1	2.3	–	3.7	Vidal-Valverde et al., 1998
	15	%	1.4	0.2	0.8	1.2	–	2.2	Lattanzio et al., 1986
Lupins	1	%	2.6	0.8	4.1	0.5	–	5.9	Sosulski et al., 1982
	51 (*L. albus*)	g kg^–1^	29.2	9.5	65.7	11.3	–	86.5	Trugo et al., 1988
	12 (*L. mutabilis*)		23.7	24.7	84.9	10.5	–	120.1	
	12 (*L. luteus*)		17.1	12.2	48.5	40.8	–	101.5	
	1 (*L. angustifolius*)		34.3	14.5	52.1	19.8	–	86.4	
	1 (*L. hispanicus*)		7.4	9.2	65.8	17.6	–	92.6	
	1 (*L. conseninii*)		26	9.3	48.9	8.8	–	67.0	
Lima bean	1 (red)	mg 100 mg^–1^	0.8	0.3	2.8	0.2	–	3.4	Oboh et al., 2000
	1 (white)		0.8	0.3	3.2	0.2	–	3.6	
	1	%	18.5	0.5	2.8	0.3	–	3.8	Sosulski et al., 1982
Black gram (Urd bean)	1	mg g^–1^	14.6	Traces	8.9	34.4	–	43.3	Reddy and Salunkhe, 1980
	24	mg g^–1^	–	0.2–8.1	8.9–37.3	14.0–31.0	–	26.6–61.6	Souframanien et al., 2014
	–	%	0.7–1.5	0.0–1.3	0.9–3.0	3.4–3.5	–	4.3–7.8	Asif et al., 2013
Mung bean	–	%	0.2–0.3	0.3–2.6	1.2–2.8	1.7–2.8	–	3.2–8.2	Asif et al., 2013
	Raw	%	1.8	0.3	1.5	2.7	–	4.5	Åman, 1979
	Germinated (72 h)		3.8	Traces	Traces	Traces	–	Traces	
	1	%	1.0	0.2	1.0	1.8	–	3.3	Sosulski et al., 1982
	Raw (cv. Giza-1)	g 100 g^–1^	–	0.4	1.5	–	–	1.9	Mubarak, 2005
	Germinated (cv. Giza-1)		–	0.0	0.0	–	–	0.0	
Pigeonpea	–	%	2.7	1.0–1.1	2.7–3.0	4.0–4.1	–	7.7–8.2	Asif et al., 2013
	Brown	mg 100 mg^–1^	1.2	0.4	0.9	1.1	–	2.3	Oboh et al., 2000
	Cream		1.7	0.6	–	1.6	–	3.5	
Cowpea	–	%	1.8–3.1	0.4–1.2	2.0–3.6	0.6–3.1	–	3.0–7.9	Asif et al., 2013
	1	%	2.6	0.4	4.4	0.5	–	5.5	Sosulski et al., 1982

### Peas

Considerable variation in the total α*-galactoside* concentration and composition exists in pea cultivars (Sosulski et al., 1982; Jones et al., 1999; Vidal-Valverde et al., 2003; Asif et al., 2013). In a study of 18 pea cultivars, total α*-galactosides* concentration ranged from 22.6 to 63.4 g kg^–1^ of dry matter (Vidal-Valverde et al., 2003). Stachyose and verbascose contents were higher compared to raffinose content (Jones et al., 1999; Vidal-Valverde et al., 2003; Asif et al., 2013).

### Soybean

Sucrose was the major sugar found in the 241 soybean plant introductions (PIs), ranging from 1.6 to 95.4 mg g^–1^, with most of the germplasm containing 30 to 70 mg g^–1^ sucrose (Hou et al., 2009a). The raffinose content in 241 PIs ranged from 0.1 to 19.9 mg g^–1^ with a mean of 8.3 mg g^–1^, with most of the germplasm containing 5 to 10 mg g^–1^. The total sugar content in 241 PIs followed a normal distribution ranging from 16.4 to 190.1 mg g^–1^, with a mean of 96.4 mg g^–1^. In another study, Hymowitz and Collins (1974) and Hartwig et al. (1997) found that raffinose was a minor sugar, whereas stachyose was the second major sugar after sucrose. The stachyose content ranged from 0.2 to 69.6 mg^–1^ with a mean of 31.7 mg^–1^ and the majority having 30 to 40 mg^–1^ (Hymowitz and Collins, 1974; Hartwig et al., 1997). The total sugar concentration is in the range of 70 to 120 mg g^–1^ (Hymowitz and Collins, 1974; Hartwig et al., 1997).

The RFOs found in most soybean cultivars were stachyose and raffinose. They made up to approximately 4–6% of soybean flour on a dry weight basis (Grieshop et al., 2003). Kennedy et al. (1985) reported that the coefficient of variation for the soybean samples were 3.5%, 5.3%, and 10.5%, respectively, for sucrose, stachyose, and raffinose. The average raffinose and stachyose content in the defatted soy flour was 1.15% and 3.23%, respectively. In soybean, the reduction in *raffinose synthase* enzyme activity was reported in the developing seeds, which means the developing seeds accumulate more sucrose than RFOs (Hitz et al., 2002).

The total α*-galactosides* concentration in soybean was 6.0 to 8.0 g 100 g^–1^ with stachyose as the major RFO (Sosulski et al., 1982). RFO accounted for more than 50% of total soluble sugars in cowpea and soybean. Giannoccaro et al. (2008) quantified the major sugars, including glucose, fructose, sucrose, raffinose, and stachyose in five soybean lines. The amount of each sugar five soybean lines ranged 0.07–0.15% for glucose, 0.08–0.19% for fructose, 5.64–9.39% for sucrose, 0.25–1.35% for raffinose, and 0.29–6.33% for stachyose.

Kumar et al. (2010) examined the ranges of sucrose and total RFO content in 48 soybean genotypes using the enzymatic rapid assay method. Sucrose content ranged from 3.45 to 16.55 mmol 100 g^–1^ with a mean value of 8.90 mmol 100 g^–1^. Total RFO content varied from 3.5 to 9.22 mmol 100 g^–1^ with a mean value of 6.64 mmol 100 g^–1^. Skoneczka et al. (2009) reported the stachyose content in wild-type and mutant types. The stachyose content for the wild type and mutant line ranged from 0.56 to 0.89% and 0.69 to 1.47%, respectively. Trugo et al. (1995) reported a range of 0.4 to 1.4 and 4.8 to 6.9 mmol 100 g^–1^ for raffinose and stachyose, respectively, in the 20 Brazilian soybean genotypes.

### Chickpea

Raffinose family oligosaccharides and sucrose are the major soluble carbohydrates found in chickpeas. The RFOs includes raffinose, stachyose, verbascose, and ciceritol. It was found that stachyose and raffinose accounted for 27.3% and 7.7% of total soluble sugars in chickpea (Lineback and Ke, 1975). Singh et al. (1982) recorded the concentrations of total soluble sugars and oligosaccharides in eight Desi and seven Kabuli type chickpea cultivars grown in Hissar, India. They found stachyose and raffinose accounted for 26.7% and 10.2% of the total soluble sugars in those varieties. Similarly, Saini and Knights (1984) studied the variation for total oligosaccharides in seven desi and Kabuli chickpea varieties. They revealed that Kabuli chickpeas had 3.2% higher total oligosaccharide levels than desi varieties. Kabuli chickpeas had 1.47, 5.30, and 0.12 g 100 g^–1^ of raffinose, stachyose, and verbascose, respectively: desi chickpeas had 1.48, 5.06, and 0.15 g 100 g^–1^ of raffinose, stachyose, and verbascose, respectively (not presented in the table). A study by Mulimani and Ramalingam (1997) reported a higher raffinose concentration (1.9 to 2.8 g 100 g^–1^ dry matter) than the total amount of stachyose and verbascose concentration (0.9 to 1.7 g 100 g^–1^ dry matter) (not presented in the table).

**FIGURE 2:**
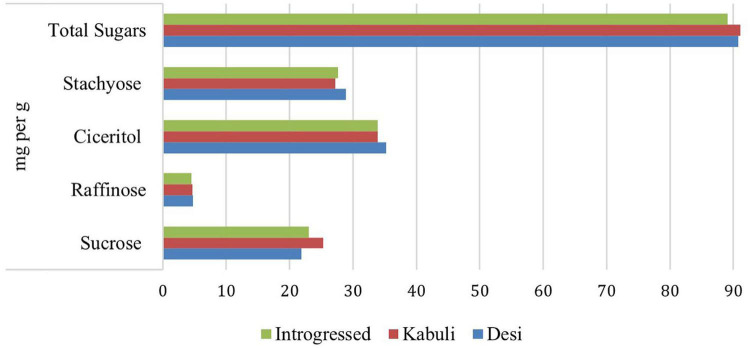
Oligosaccharide variations in the ICRISAT chickpea mini-core collection.

The chromatographic profile of three raw Spanish chickpea cultivars was observed and the soluble sugar content was quantified by Sánchez-Mata et al. (1998). Among disaccharides, sucrose was the leading one, accounting for 28% of the total sugar content. The α*-galactoside* group was around 60% of the total sugar content in raw samples. Ciceritol comprised about 40% of total sugars in chickpea samples analyzed. Other α*-galactosides* in total, when analyzed in raw chickpea, ranged from 1.31 mg 100 g^–1^ to 1.95 mg 100 g^–1^ and represented 19.4% to 22.3% of the total amount of sugars. Figure 2 captures the oligosaccharide variation present in the International Crops Research Institute for the Semi-Arid Tropics (ICRISAT) mini-core collection of chickpeas (Ramadoss et al., 2015). Kabuli chickpeas had higher total sugars and sucrose levels, whereas desi had higher levels of RFOs, including raffinose, stachyose, and ciceritol (Figure 2). Germination considerably reduced the accumulation of RFOs in chickpea seeds (Åman, 1979), and according to Xiaoli et al. (2008), chickpea sample 171 has the desirable sugar profile and could be used in breeding programs to develop ideal sugar types.

### Lentil

Significant variations were observed for α*-galactosides* in lentil seeds ranging from 1.8 to 7.5% (Wang and Daun, 2006; Martínez-Villaluenga et al., 2008). Sucrose concentration of lentil cultivars ranged from 1.2 to 1.7 g 100 g^–1^ flour with a mean concentration of 1.4 100 g^–1^ flour. Total RFO concentration of lentil cultivars varied from 4.5 to 5.5 moles 100 g^–1^ flour. Johnson et al. (2021) found 7 to 31% variation for RFOs in lentil seeds from 143 accessions. Significant differences were observed between different years of lentil harvest. The year 1995 harvest exhibited higher total α*-galactosides* compared to the year 1994 harvest (Sánchez-Mata et al., 1998), which explains the accumulation of soluble sugars is highly dependent on the environment and tissue and is genotype-specific (Sánchez-Mata et al., 1998; Han and Baik, 2006; Martínez-Villaluenga et al., 2008).

### Faba Bean

Sosulski et al. (1982) studied variation in the concentration of α*-galactoside* in 11 legumes and reported verbascose is the predominant α*-galactoside* in faba bean. Vidal-Valverde et al. (1998) observed high levels of verbascose (2.29% of dry weight), followed by stachyose (1.10% of dry weight) and raffinose (0.28% of dry weight) in faba bean. Lattanzio et al. (1986) reported the oligosaccharides content in fresh and dry mature seeds of fifteen cultivars of faba bean lines. The raffinose content of the whole dry seeds ranged from 0.12 to 0.29%; stachyose content between 0.46 and 1.02%; and verbascose content between 0.82 and 1.61% on a dry matter basis.

### Lupins

A wide variation for RFO concentration and its composition was reported among the lupin species: it had 0.30–1.90, 2.30–8.60, and ND (non-detectable)–3.50 percent of raffinose, stachyose, and verbascose, respectively (Martínez-Villaluenga et al., 2008). Ajugose had an exclusive presence in lupin seeds: *L. albus*, *L. mutabilis* had the lowest level of ajugose (0.2–0.5% and 0.2%, respectively), followed by *L. angustifolius* (1.7–2.6%) and *L. luteus* (0.6–4.6%) (Trugo et al., 1988). Trugo et al. (1988) studied the RFO content and sucrose in various lupin species. They found significant variations in the levels of individual RFO among lupin species. *L. albus* seeds had the verbascose (0.4%); *L. luteus* had stachyose (7.4%), verbascose (3.1%), sucrose (1.2%), and *L. angustifolius* had sucrose (3.4%) and stachyose (4.6%). There was a wide variation in total α-galactosides between species, with a remarkably high content found in *L. luteus* (9.5–12.3%).

### Lima Beans

The effect of oligosaccharides on germination was investigated in lima beans (Dibofori et al., 1994). The sucrose content increased from 190 mg 100 g^–1^ and 790 mg 100 g^–1^ by the fifth day of germination. On the other hand, raffinose decreased from 620 mg 100 g^–1^ to 131 mg 100 g^–1^ on the fifth day of germination. Oboh et al. (2000) reported the total α*-galactoside* contents of the seeds in the mature seeds in white lima beans (3.62 mg 100 mg^–1^) and red lima beans (3.37 mg 100 mg^–1^). They found stachyose was the predominant sugar in lima beans.

### Black Gram (Urd Bean)

Reddy and Salunkhe (1980) reported RFO concentration in long-grain polished rice and black gram. They did not find RFO in rice but higher verbascose concentration (3.44%), followed by stachyose (0.89%) and raffinose (trace) in black gram. Souframanien et al. (2014) also reported higher verbascose concentration (14 to 31 mg g^–1^), followed by stachyose (8.9 to 37.3 mg g^–1^) and raffinose (0.2 to 8.1 mg g^–1^) in black gram.

### Green Gram (Mung Bean)

Verbascose was the prominent RFO sugar in green gram (Åman, 1979; Sosulski et al., 1982; Asif et al., 2013). Germination completed nullified the presence of RFOs in green gram compared to the raw seeds (Åman, 1979; Mubarak, 2005). Hence, consuming sprouted seeds of mung bean is the effective alternate strategy to avoid flatulence problems in human beings.

### Other Grain Legumes

The raffinose (3.7 mg g^–1^) and stachyose (23.6 mg g^–1^) contents were reported in dry bean varieties (Sosulski et al., 1982). Dry bean varieties grown in the United States have about 2–10 mg g^–1^ of raffinose and 2–56.2 mg g^–1^ of stachyose (Salunkhe and Kadam, 1989). Oboh et al. (2000) quantified the total α*-galactoside* contents in the mature seeds of pigeon peas, African yam beans, and jack beans. The total α*-galactoside* contents of the seeds in decreasing order were African yam beans (3.84 mg 100 mg^–1^); cream pigeon peas (3.52 mg 100 mg^–1^); jack beans (2.83 mg 100 mg^–1^), and brown pigeon peas (2.34 mg 100 mg^–1^). Stachyose was the predominant sugar in jack beans and African yam beans, while verbascose was the predominant oligosaccharides in pigeon pea. Stachyose was the prominent sugar in cowpea (Sosulski et al., 1982; Asif et al., 2013).

## Metabolism and Genetics of Raffinose Family Oligosaccharides

Raffinose family oligosaccharides are α-D-galactosides of sucrose, a di-saccharide. They also occur in forms such as raffinose, stachyose, verbascose, and ajugose, belonging to trisaccharide, tetrasaccharide, pentasaccharide, and hexasaccharide groups, respectively (Bachmann and Keller, 1995; Vanhaecke et al., 2006, 2008, 2010). From the structural perspective, they are considered α-galactosyl derivatives of sucrose. Raffinose contains galactose, glucose, and fructose. Stachyose holds two α-D-galactose units, one α-D-glucose unit, and one β-D-fructose unit. Besides *galactinol synthase*, *raffinose synthase* and *stachyose synthase* are the other two major enzymes involved in the RFO biosynthesis pathway. *Raffinose synthase* transfers a galactinol moiety from galactinol to sucrose and produces raffinose, while the *stachyose synthase* utilizes galactinol to synthesize tetrasaccharide stachyose. Both reactions are reversible (Sengupta et al., 2015). However, the enzyme responsible for the biosynthesis of verbascose has not been recognized yet (Lahuta et al., 2010). Peterbauer et al. (2003) reported that *stachyose synthase* is the most probably responsible enzyme due to its multifunctional character. Recently, Kannan et al. (2018) have noted that the *verbascose synthase* is the chief enzyme that catalyzes the synthesis of verbascose by adding a galactosyl residue from galactinol to stachyose. Nevertheless, the synthesis of the pentasaccharide verbascose and higher homologs is galactinol-dependent. They may include α-galactosyl derivatives of the cyclitols myo-inositol, D-pinitol, D-chiro-inositol, and D-ononitol but with small amounts (Obendorf and Górecki, 2012).

Raffinose family oligosaccharides are ubiquitous and can be directly extracted from the plant materials using water or aqueous ethanol, or methanol solutions. Raffinose is mainly extracted from sugar beets during sugar processing in Japan (Dinoto et al., 2006). But traditional methods of extraction processes often yield low and fetch high production costs (Han et al., 2019). Therefore, high throughput, a cost-effective, environment-friendly method is imperative. Several researchers have focused on the production of α*-galactosidase* using microorganisms, which offer great potential for enzyme production due to high expression levels and extracellular secretion into the medium, promoting easier downstream processing. Filamentous fungi were the most sought microbial source exploited extensively for the synthesis of α*-galactosidase* and have been used in multiple biotechnological and medical applications (Katrolia et al., 2014). Numerous investigations have been reported the production of α*-galactosidase* originated from several fungi such as *Aspergillus niger*, *Aspergillus parasiticus*, *Cladosporium cladosporides*, and *Aspergillus niger* (Mansour and Khalil, 1998), and *Pestalotiopsis microspora* (Yang et al., 2015). Likewise, the α*-galactosidase* gene has been characterized from many bacterial sources such as *Bacillus stearothermophilus* (Gote et al., 2004, 2006) and *Thermotoga maritima* (Comfort et al., 2007). Fridjonsson et al. (1999) isolated the enzyme designated *AgaN*, a similar gene to α*-galactosidase* from *Bacillus stearothermophilus.* The enzyme showed high thermostability and displays a high affinity for oligomeric substrates, including the raffinose, and can hydrolyze raffinose in the presence of 60% sucrose with high efficiency.

Recently, Huang et al. (2018) characterized a novel α*-galactosidase* (*AgaB*) from *Bacillus megaterium* and exhibited high activity in the intestine. The *AgaB* gene completely hydrolyzed raffinose and stachyose and rapid hydrolysis of RFO in soybean milk at 37°C within 4 h when combined with trypsin. Likewise, *Aga-BC7050* is a novel α*-galactosidase* of glycoside hydrolase family cloned from *Bacillus coagulans* and was highly active toward raffinose and stachyose (Zhao et al., 2018). *Aga-BC7050* showed great resistance to proteinase and trypsin, not inhibited by monosaccharides, and completely hydrolyzed raffinose and stachyose in less than 30 min. Furthermore, two genes (*agal1* and *agal2*) encoding α*-galactosidase* were identified by sequence-based screening approaches from two thermophilic bacteria (Schröder et al., 2017). Qiu et al. (2015) identified and characterized a mutant gene of soybean stachyose (*STS*) gene controlling the reduction of stachyose and raffinose content up 90% in soybean seeds also confirmed the function of *STS* gene in converting raffinose into stachyose as part of raffinose metabolism.

Utilization of altered *raffinose synthase 2* (*RS2*) alleles is the straightforward genetic approach to high sucrose and low RFOs trait. The enzyme *RS2* is *galactinol-sucrose galactosyltransferase* and is considered the committed step in the biosynthesis of raffinose and the free cyclitol myo-inositol. In soybean, variant alleles of the *RS2* gene have been identified and showed a high capacity in decreasing RFOs and increasing sucrose content, which may improve metabolizable energy in soybean meal (Dierking and Bilyeu, 2008, 2009; Skoneczka et al., 2009). Bilyeu and Wiebold (2016) demonstrated that altered carbohydrate soybeans produce high sucrose (>8% dry) and low RFOs (1% total raffinose and stachyose) phenotype across different environments with contrasting alleles of the *RS2* gene. Similarly, Valentine et al. (2017) used RNA-mediated gene silencing to down-regulate the soybean gene *RS2*. They identified increased true metabolizable energy (from 2.41 to 2.70 kcal kg^–1^) in poultry feed using transgenic soybean lines, which exhibited intensely reduced raffinose levels in mature seed. *RS3* and *RS4* genes with polymorphisms that contributed to the ultra-low raffinose and stachyose content and identified a novel mutant allele designated *SG-ULRFO* derived from soybean, which resulted in an ultra-low raffinose and stachyose phenotype (Schillinger et al., 2013). Hagely et al. (2020) suggested that the combination of *RS2* and *RS3* alleles produce ultra-low phenotypes in soybean. On the other hand, a strong relationship between RFOs and abscisic acid (ABA) in mature seeds of alfalfa, where the amount of galactinol, raffinose, and stachyose accumulated was much higher (> threefold) at the highest ABA concentration (Blöchl et al., 2005). This accumulation was attended by a threefold increase in *galactinol synthase* activity, while the levels of *raffinose synthase* and *stachyose synthase* activities persisted almost constant. In addition, high ABA applications declined the content of monosaccharides (glucose and fructose).

On the other hand, α*-galactosidases* are classified into several glycoside hydrolases (GH) families, such as GH4, GH27, GH36, and GH57. However, most characterized α*-galactosidases* are assigned into the evolutionary related GH27 and GH36, which together with GH31 represent clan GH-D comprising a common fold and handling double displacement mechanism (Comfort et al., 2007). Most GH36 α*-galactosidases* were characterized as tetramers, often larger enzymes of about 85 kDa (Garro et al., 1993; Nakai et al., 2010). A new gene encoding a putative α*-galactosidase* (*CbAga36*) from *C. bescii* was cloned and sequenced (Lee et al., 2017). The size exclusion chromatography indicated that its native form was a tetramer. *CbAga36* was grouped as members of the clan GH-D, exhibiting high similarity to the family of glycoside 36. Besides, the purified recombinant of *CbAga36* demonstrated preferential activity toward the hydrolysis of RFO, and the enzyme activities were stable at high-temperature levels ranging from 60 to 75°C. The inheritance pattern of total α*-galactoside* and individual RFO compounds and ciceritol were determined using embryos and the seed coat from single seeds of the reciprocal crosses (Frias et al., 1999). Within α*-galactosides*, raffinose was usually present in embryos in the lowest amount, not varying between the parental, F_1_, and F_2_ generations. However, a wider variation was found between the ten F_3_ families, ranging from 0.3 to 0.6%. Stachyose formed a significant proportion of the total α*-galactosides* found in all embryos. The mean values for the parental lines were similar to each other (2.3% and 2.5%, respectively) and the level found in reciprocal. The widest variation between the parental lines was found for verbascose content, ranging from 0.6 to 1.0%.

## Role of Raffinose Family Oligosaccharides in Plant Health

The importance of RFOs in plant health is still an area of interest for researchers; and has many gaps to fill even after years of study. Till now, it is known that they have a crucial role in plant physiology and cellular functions as signal transduction (Stevenson et al., 2000; Xue et al., 2007), membrane trafficking, mRNA export and transport, carbon storage (Thole and Nielsen, 2008; Okada and Ye, 2009), desiccation tolerance (Martínez-Villaluenga et al., 2008), seed storability (Horbowicz and Obendorf, 1994), biotic and abiotic stress tolerance (Nishizawa et al., 2008; Nishizawa-Yokoi et al., 2008), photoassimilate translocation (Dinant and Lemoine, 2010), and seed germination (Blöchl et al., 2007). The role of RFOs in plant health is further discussed below.

### Carbon Storage and Translocation

Plants store and translocate fixed carbon anticipating the worst fluctuation in the environmental conditions, which may eventually reduce carbon supply. Most of the time, they store carbon in the form of starch while translocating them in sucrose. However, certain plant species can store and translocate alternate carbohydrates like RFOs (Kandler and Hopf, 1982). For example, the plant family members; *Cucurbitaceae*, *Lamiaceae*, *Oleaceae*, and *Scrophulariaceae* transports RFOs through phloem by forming a symplasm with mesophyll and sieve elements (Beebe and Turgeon, 1992; van Bel, 1993; Turgeon, 1996).

### Abiotic and Biotic Stress Tolerance

Even though RFOs are derived from an extended metabolic pathway of inositol, they don’t directly involve in plants’ stress amelioration under natural conditions, unlike other products derived from the same pathway (Loewus and Murthy, 2000; Sengupta et al., 2012). A subsequent increase of RFOs (especially raffinose) has been observed in several cases of abiotic stresses such as heat, cold, salinity, or drought (Santarius and Milde, 1977; Bachmann et al., 1994; Taji et al., 2002; Pennycooke et al., 2003; Panikulangara et al., 2004; Nishizawa-Yokoi et al., 2008; Peters and Keller, 2009; Peters et al., 2010). However, there is not much information explaining the specific functional roles of RFOs in abiotic stress tolerance. Several other molecules (e.g., sucrose and proline) with characterized roles in abiotic stress amelioration also tend to accumulate under such conditions. Reports have also suggested that genetic elimination of biosynthetic enzymes associated with RFOs does not affect plants drastically (Panikulangara et al., 2004), adding further evidence to the above fact. On the contrary, certain studies claim that RFOs do have beneficial properties of a compatible solute. For example, research by Hincha et al. (2003) suggests that RFOs stabilize the cell membrane during dehydration stress by inserting themselves within the lipid head groups of the membrane bilayer. Farrant (2007) added further proof to this fact by correlating the phenomenon of increase in RFOs during desiccation and stabilization of membrane phospholipids. Moreover, their high oligomeric length may positively impact protecting liposomes (Cacela and Hincha, 2006) and possibly act as a free radical scavenger (Nishizawa-Yokoi et al., 2008). Furthermore, several reports suggest that the accumulated RFOs under abiotic stress conditions function as osmolytes to maintain cell turgor and act as an antioxidant against reactive oxygen species (Nishizawa-Yokoi et al., 2008; van den Ende and Valluru, 2008; Bolouri-Moghaddam et al., 2010; Stoyanova et al., 2011; van den Ende et al., 2011; Peshev et al., 2013).

*Galactinol synthase* (*GolS*) is a key enzyme that is involved in the biosynthesis of RFOs (Saravitz et al., 1987) and is known to be linked to abiotic stress (Sengupta et al., 2015). Therefore, genetically modulating the expression of *GolS* genes can provide much information about the involvement of RFOs in mediating response to abiotic stresses. These studies have been carried out mainly in *Arabidopsis thaliana* or tobacco (*Nicotiana tabacum*) plants, as they seem to elevate galactinol and raffinose content in response to abiotic stresses (Taji et al., 2002; Zhuo et al., 2013; Himuro et al., 2014; Shimosaka and Ozawa, 2015; Gu et al., 2016). Multiple isoforms of *GolS* have been identified from various plant species so far; each is synthesized under various circumstances of abiotic stresses. It has been found that out of seven identified *GolS* genes from *Arabidopsis thaliana*, *AtGolS1* and *AtGolS2* were induced by drought, salt, or heat stress. In contrast, *AtGolS3* from the same genome were induced by cold stress (Taji et al., 2002). Over-expressing or knocking out these genes can be made use of for the study of RFOs physiology. Studies by Taji et al. (2002) and Panikulangara et al. (2004) showed that over-expression of these genes induced accumulation of galactinol (*Gol*), raffinose (*Raf*), and stachyose (*Sta*) and eventually improved the plant’s tolerance level to drought, salinity, or cold stress. Panikulangara et al. (2004) also proved that *AtGolS1* mutant plants fail to accumulate heat stress-induced *Gol* and *Raf*, indicating that *AtGolS1* may be the crucial *GolS* isoform responsible for heat stress-induced *Raf* or *Gol* accumulation. However, a study by Peters et al. (2010) involving a double mutant; claimed that despite the improved accumulation of *GolS1* in *GolS2* mutants, they remain hypersensitive to water stress, exhibit rapid loss of water and lower enzymatic activity. Hence indicating they are drought hypersensitive. Such observations concerning the fact that *Arabidopsis* neither stores nor transports RFOs prove the involvement of various biosynthetic pathways that are supplied to by different *GolS* isoforms. Likewise, cold temperature tolerance was achieved by overexpressing the *Medicago falcata Gols* (*MfGolS1*) gene in tobacco (Zhuo et al., 2013).

**FIGURE 3:**
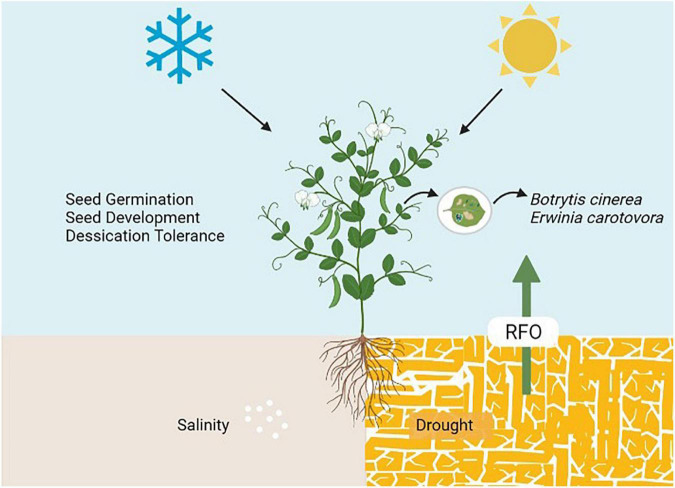
Multi-functional role of RFOs in plant health.

Valluru and van den Ende (2011) explained the role of galactinol in signaling RFOs to mediate stress responses, including a signal in response to pathogen infection. Thus, evidencing the role of RFOs in defense against biotic stress. *GolS* induced the expression of defense-related genes such as *PR1a*, *PR1b*, and *NtACS1* in tobacco upon *Botrytis cinerea* and *Erwinia carotovora* infection (Kim et al., 2008). Also, *Gol* induces the salicylic acid (SA) signaling upon pathogen infection and eventually turns on the *PR1a* gene expression to control disease progression (Couée et al., 2006). RFOs (mainly *GolS* and *RafS*) contain W-box *cis*-elements in their promotors, regulated by ABA-inducible WRKY (Wang et al., 2009). This suggests a possible role of RFOs in SA and ABA signaling under biotic and abiotic stresses. Figure 3, depicting the role of RFOs in plant health, including their role in seed germination, seed development, desiccation tolerance, biotic and abiotic stress tolerance.

### Seed Germination

Raffinose family oligosaccharides accumulate over the period in all parts of developing seeds, including endosperm, embryo, and seed coat (Kuo et al., 1988; Horbowicz and Obendorf, 1994; Frias et al., 1999). But the level and class of RFOs deposition may vary between different tissues. These RFOs significantly impact seed germination, often protect the embryos from desiccation during seed maturation and improve the longevity of the seeds under adverse conditions (Peterbauer et al., 2002a,b). During the early stages of seed germination, RFOs are readily available and provide energy and carbon to the germinating seeds (Zhao et al., 2006).

### Desiccation Tolerance

The seed germination process demands a lot of water; the water loss during seed germination is known as “desiccation.” This may lead to membrane damage and the death of the embryo. Accumulation of non-reducing sugars such as sucrose and RFOs may prevent the desiccation process in seeds (Koster and Leopold, 1988), and many reports suggested the role of RFOs in desiccation tolerance (Blackman et al., 1992; Corbineau et al., 2000; Angelovici et al., 2010). There were two mechanisms reported where RFOs act in mitigating the desiccation process in seeds. The first mechanism is known as “water replacement,” where the hydroxyl groups of RFOs can replace water molecules and maintain the hydrophilic interactions within the cell that are necessary for stabilizing native macromolecules and membrane structure during dehydration process (Koster, 1991). The second mechanism is called “vitrification.” This is the state of a cell solution having very high viscosity due to loss of water. At this state, the cell solution has the properties of a plastic solid. It is accountable for warranting stability, preventing cellular collapse, and maintaining hydrogen bonding within the cell (Koster and Leopold, 1988; Koster, 1991; Martínez-Villaluenga et al., 2008; Angelovici et al., 2010). Pukacka et al. (2009) reported that the late embryogenesis abundant (LEA) proteins and small heat shock proteins (sHSP) along with RFOs are responsible for the vitrification state.

## Role of Raffinose Family Oligosaccharides in Human Health

Raffinose family oligosaccharides are ubiquitous in legume seeds (Minorsky, 2003), and they are composed of α*-(1,6)-galactosides* linked to a sucrose unity (Cardoso et al., 2021). Humans and animals do not produce an α*-galactosidase* enzyme to synthesize and digest the RFOs in the intestine (Minorsky, 2003; Mao et al., 2014). Therefore, RFOs escape the digestion process and get utilized by the gut microbes (bacteria) to synthesize by-products like hydrogen (H_2_), carbon dioxide (CO_2_), and methane (CH_4_). Thus, RFOs primarily cause flatulence in humans and animals (Naczk et al., 1997; Minorsky, 2003). Hence, RFOs are considered the single most deterring factor for the wide acceptance of legumes in human and animal diets (Delumen, 1992). Therefore, only a limited quantity of soybean meals was allowed in animal feeds to avoid flatulence and digestive problems in dogs (*Canis familiaris*), baby pigs (*Sus scrofa*), and chickens (*Gallus domesticus*) (Hartwig et al., 1997). The pharmaceutical company *GlaxoSmithKline* released a commercial product^1^ “*Beano*,”^1^ which supplies α*-galactosidase* and *sucrase* enzymes in the human body to hydrolyze RFOs mitigate the flatulence problem. Recently, the α*-galactosidase* gene (*galC*) was cloned from *Aspergillus oryzae* (*YZ1*) and expressed in *Pichia pastoris* for protein production, and *galC* effectively degraded the RFOs (primarily raffinose and stachyose) in soymilk (Wang et al., 2020). But recent studies have shown the benefit of RFOs in human health (Dinoto et al., 2006; Fernando et al., 2010; Takagi et al., 2016; Pacifici et al., 2017; Collins et al., 2018; Ose et al., 2018; Xu et al., 2018; Zartl et al., 2018; Amorim et al., 2020; Cardoso et al., 2021). Moreover, RFOs can be converted into prebiotic molecules using enzymes via catalytic transformations. For example, *levansucrase* can convert raffinose to melibiose and stachyose to mannotriose (Park et al., 2003; Xu et al., 2017; Jadaun et al., 2019).

Recent studies reported the prebiotic potential of the RFOs in human guts; they promoted the growth of beneficial bacterias such as *Bifidobacteria* and *Lactobacilli* and reduced the harmful bacterias present in the colon (Mao et al., 2014; Takagi et al., 2016; Ose et al., 2018; Zartl et al., 2018; Amorim et al., 2020). Apart from the beneficial effect on gut microbiota, RFOs administration improved the intestine microbial composition in healthy adults (Dinoto et al., 2006; Fernando et al., 2010), improved the growth of sturgeon hybrids (Xu et al., 2018), improved the Fe availability and intestinal brush border membrane functionality in *Gallus gallus* (Pacifici et al., 2017), and RFOs increased the number of *Lactobacillus* (beneficial bacteria) present in the vaginal microbiota (Collins et al., 2018). Many other biological activities of RFOs have been reported apart from the prebiotic potential, such as anti-allergic (Nagura et al., 2002; Watanabe et al., 2004), anti-obesity, anti-diabetic, and prevention of non-alcoholic fatty liver disease through inhibition of lipid accumulation (Muthukumaran et al., 2018), reduction of fecal ammonia and indole (Nagura et al., 1999; Elliott et al., 2017), cryoprotection (Elliott et al., 2017), and inhibition of *Pseudomonas aeruginosa* biofilm formation (Kim et al., 2016).

Lupin seeds have high RFOs compared to other pulses (Martínez-Villaluenga et al., 2005; Johnson et al., 2017). The extract from lupin seeds had positive effects on the survival of probiotic cultures in dairy products (Martínez-Villaluenga et al., 2006), and RFOs isolated from the novel plants *Rehmannia glutinosa*, used as food ingredients to prevent ROS-related liver damage (Dai et al., 2018). RFOs also reduced the severity of colon inflammation in mice (Zhang et al., 2013). Raffinose isolated from the rhizome of *Costus speciosus* inhibits lipid accumulation (Muthukumaran et al., 2018), and stachyose prevents ulcerative colitis in mice (He et al., 2020). RFOs are reported to have a therapeutic effect on curing cutaneous disorders (Na et al., 2017). Largely, RFOs may have diverse applications in food (human), feed (animal), cosmetic, health (human, plant, and animal), and pharmaceutical, and received food for specified health uses (FOSHU) status in 2003 (Bailey, 2005), and widely consumed as a functional ingredient in Japan (Takakuwa et al., 2007). However, wider recognition in other parts of the world as functional foods is not yet reached.

## Emerging Research Issues

Raffinose family oligosaccharides could be exploited as functional foods. Its multi-functional benefits are still yet to be realized in human and animal well-being. RFOs positively affect the gut microbiota, large intestines, and colon health and could be used as therapeutic agents to reduce inflammation, diabetics, allergies, etc. RFOs are considered the prime suspect for flatulence in humans and animals. Hence, for the crops with high RFOs, especially grain legumes, adoption in the food and feed system is heavily impaired due to the flatulence problem. Therefore, we need to strike the right balance of RFO content in crops to promote them as functional foods. Still, the right concentration of RFOs needed for human well-being is the area to be explored further. Moreover, except Japan, other parts of the world have yet to approve RFOs as functional foods.

Achieving desirable sugar profiles without compromising yield, protein, oil, and other micronutrients in grain legumes is important to satisfy the changing dietary habits. A significant negative correlation was reported between protein content and RFOs in soybean (Hartwig et al., 1997; Bueno et al., 2018). But a significant positive correlation was reported between oil and RFO content in soybean (Patil et al., 2017; Bueno et al., 2018). We need to fine-tune the portion of protein, fat, sugar, and oil contents in grain legumes through breeding by developing genotypes with high yield and balanced nutrition. Recently, CRISPR knockouts helped reduce the RFO contents considerably in soybean, including the major RFO sugar stachyose, by 35%. But raffinose content was increased to 42%, and protein and fat contents were also increased (Le et al., 2020).

Future research must focus on mitigating the issues related to quality and soluble sugars in legumes. Sprouting was reported to reduce the RFO contents in seeds in various legumes (Åman, 1979; Raman et al., 2019). Reduction of *myo-inositol* level led to a drastic decrease of *galactinol* and RFO levels in mutant soybean seeds (Hitz et al., 2002). Mutations in *myoinositol phosphate synthase* may affect RFO concentration, and they are potential targets for modifications of RFO content in plants. There is an urgent need to understand the relationship between *galactinol synthase* activity and the accumulation of RFO in plant tissues, which would be another exciting area of research. Finally, fine-tuning the optimum level of RFOs in crop plants to reap the benefits of RFOs in plant and human health is yet to be explored further.

## Author Contributions

DE conceived the need for the review. DE, KR, LV, SS, NT, NE, and BR jointly wrote the first draft. JR, WW, AF, KC, MT, RV, and SC edited the first draft. AS and AKS edited and provided guidance to finish the final draft for submission. All authors read, edited and approved the manuscript.

## Conflict of Interest

The authors declare that the research was conducted in the absence of any commercial or financial relationships that could be construed as a potential conflict of interest.

## Publisher’s Note

All claims expressed in this article are solely those of the authors and do not necessarily represent those of their affiliated organizations, or those of the publisher, the editors and the reviewers. Any product that may be evaluated in this article, or claim that may be made by its manufacturer, is not guaranteed or endorsed by the publisher.

## Footnotes

1
www.beanogas.com
